# Physiological Responses of *Populus cathayana* and *Salix babylonica* to Combined Stress of Diesel Fuel and Sr^2+^ Stress in Soil

**DOI:** 10.3390/plants13243598

**Published:** 2024-12-23

**Authors:** Chunyan Luo, Tingting Jiang, Peng Ren, Zhirong Suo, Ke Chen

**Affiliations:** 1 School of Life Science and Engineering, Southwest University of Science and Technology, Mianyang 621010, China; chunyan_luoo@163.com (C.L.); jiangtt1210@163.com (T.J.); renpeng@swust.edu.cn (P.R.); 2 Analytical and Testing Center, Southwest University of Science and Technology, Mianyang 621010, China; 3 Aerospace Planning and Design Group Co., Ltd., Yunnan Branch, Kunming 650216, China

**Keywords:** combined stress, Sr^2+^ stress, diesel stress, tree physiology, *Populus*, *Salix*

## Abstract

Diesel spills and nuclides pollution cause global ecosystem and human health problems. The remediation of contaminated soil using woody plants has received considerable attention. Differences in plant species and sex can lead to differences in tolerance to various stressors. We aimed to investigate the response of male and female seedlings of *Populus cathayana* and *Salix babylonica* to diesel and Sr^2+^ stress and to compare the enrichment characteristics of Sr^2+^ in trees. Male and female seedlings of *P. cathayana* and *S. babylonica* were treated with diesel fuel and 0, 10 (low), and 100 (high) mg Kg^−1^ of Sr^2+^. Results showed that *P. cathayana* and *S. babylonica* had good enrichment characteristics and tolerance. *S. babylonica* had a more robust tolerance and ability to remediate contaminated soil than *P. cathayana*. The defense mechanisms of both female seedlings in response to stress were similar, while males showed different defense strategies. Male trees had higher Sr^2+^ enrichment capacity, antioxidant enzymes, soil enzyme activity, and soluble matter content, indicating that males had higher tolerance capacity than females. Under diesel stress alone, the reduced photosynthetic rate of male seedlings of *P. cathayana* was mainly limited by stomatal factors, and their photosynthetic system was more tolerant to diesel. POD and APX activities, as well as alkaline phosphatase and urease activities in the soil, were significantly higher in *S. babylonica* seedlings than in *P. cathayana*, indicating that *S. babylonica* seedlings were more resistant to diesel pollution. At low concentrations of the Sr^2+^ complex, diesel and Sr^2+^ showed antagonistic effects in reducing the damage caused by stress. As the Sr^2+^ concentration increased, damage to the plants manifested primarily through synergistic enhancement. The results of this study provide a scientific basis for the remediation of diesel fuel and nuclides contaminated soils using woody plants.

## 1. Introduction

Nuclide metal toxicity is a major problem in the natural environment due to the non-biodegradable properties and persistence of nuclides in nature [1]. Strontium (Sr) is a common alkaline nuclide earth metal used extensively in industries, including fireworks and electronics (e.g., historically in cathode-ray televisions); it is also a contaminant resulting from nuclear accidents, as in the Chernobyl (1986) and Fukushima (2011) nuclear disasters [2]. More recently, strontium concerns resurfaced as a naturally occurring geochemical contaminant from tertiary petroleum and natural gas extractions. Owing to their high solubility in water, they easily spread to nearby soil and water, eventually contaminating aquatic ecosystems and foodstuffs, which enter the human body in this way [3]. Sr^2+^ can be deposited in human bone tissue, teeth, liver, and other organs, especially during abnormal bone development in children [4]. Some studies have reported that the strontium in soil conditions was able to impact plants, including productivity, growth, and development. For example, Zheng et al. studied the stress response of mosses to Sr^2+^ air pollution and found that significant Sr^2+^ adsorption led to a decrease in plant biomass and chlorophyll content, yellow leaf death, and an increase in leaf plasma membrane permeability [5]. In addition, chronic oral toxicity values from flowback waters that included Sr were also reported [6]. Sowa et al. showed that plants can efficiently accumulate Sr^2+^, and young soybean seedlings can efficiently absorb Sr^2+^ and transfer it to the aboveground parts of the plant, where the concentration of Sr^2+^ is approximately 7–9 times higher than that of the roots [7]. Currently, phytoremediation technology is mainly adopted to treat organic matter pollution, such as heavy metals, nucleophiles, and polycyclic aromatic hydrocarbons, in the soil [8].

Around the world, large areas of soil and water reservoirs have been contaminated with oil products such as diesel fuel. Contamination of soil by oil spills is a widespread environmental problem that often requires cleaning up of the affected site [9]. Diesel fuel is one of the derivative forms of petroleum that is widely used in the world. Its composition has many aromatic compounds and long hydrocarbon chains, both persistent and hazardous. Of the components in diesel fuels, compounds of lower molecular mass will tend to evaporate and degrade more readily; therefore, the harmful effects of the release of PAHs from diesel fuels into the soil are important. Diesel fuel and nuclide metals have different physicochemical properties, and various interactions occur when they coexist in the soil, mainly ionic interactions, competition for adsorption sites, and redox reactions [10]. When the amount of diesel fuels and nuclides in the soil exceeds the self-purifying capacity, it causes compound soil pollution, and remediation of compound soil pollution is more difficult than remediation of single contaminated soil. Pollutants eventually accumulate in sediments, organic matter, and organisms, thus posing a threat to human health and the entire ecosystem [11]. Therefore, remediation of soils contaminated with diesel fuel and nuclides complexes has become an interesting research topic.

Poplar and willow trees are considered promising candidates for the remediation of soil contaminated with diverse pollutants because of their perennial nature, fast growth rate, high biomass, well-developed root system, and high resistance to adversity [12]. Recent studies have shown that dioecious plants, such as poplars, exhibit sex differences in their defense responses to abiotic stresses, with males usually being more tolerant than females [13]. In general, sex differences are interpreted as the result of different reproductive functions, and sex dimorphism in physiological characteristics results from the different reproductive needs of females and males, leading to sex-specific selection pressures [14]. Females allocate more reproductive resources than males to flower, fruit, and seed production. Males are usually associated with more nutritional resources for growth and tolerance to abiotic stress than females [15,16]. Chen et al. found that females of the Yunnan poplar were more sensitive to Cd stress than males [17]. Compared to herbaceous plants, woody plants have the advantages of well-developed root systems and higher biomass, generally do not enter the food chain, and are long-lived. Therefore, woody plants are increasingly being used to remediate contaminated soils [18].

In this experiment, diesel fuels and the nuclide metal Sr^2+^ were used as pollutants in a pot test on male and female seedlings of *P. cathayana* and *S. babylonica*. This study of the stress mechanism of diesel and Sr^2+^ in different species of trees provides a theoretical basis for the use of phytoremediation techniques to manage nuclides and diesel fuel complex pollution. Therefore, it is essential to research the composite pollution of nuclides and organic matter for ecological environmental protection and management.

## 2. Results

### 2.1. Sr^2+^ Enrichment in Tissues of P. cathayana and S. babylonica

**Figure 1:**
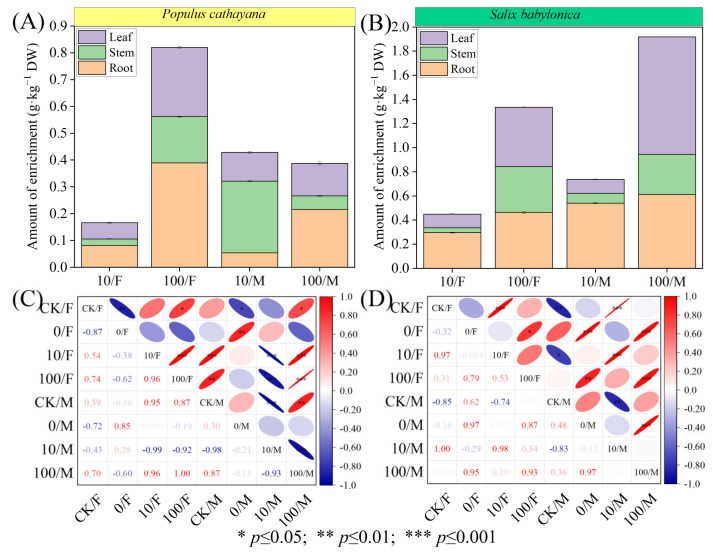
Sr^2+^ enrichment and correlation analysis between different tissues of female and male seedlings of *P. cathayana* and *S. babylonica*. Sr^2+^ enrichment in root, stem, and leaf tissues of *P. cathayana* (**A**) and *S. babylonica* (**B**); correlation coefficients between different treatment groups of *P. cathayana* (**C**) and *S. babylonica* (**D**).

The data showed that the enrichment capacity of *S. babylonica* for Sr^2+^ was much higher than that of *P. cathayana* (Figure 1A,B). Under the compound stress of Sr^2+^ at low concentrations, the enrichment concentration of Sr^2+^ in male seedlings of *P. cathayana* was stem > leaf > root. In the other seedlings, it was root > leaf > stem, and the enrichment concentration of Sr^2+^ in male seedlings of both plants was higher than that in male seedlings. Under the high concentration of Sr^2+^ complex stress, the concentration of Sr^2+^ enrichment in *P. cathayana* female and male seedlings was root > leaf > stem, and the enrichment in *S. babylonica* seedlings both showed leaf > root > stem. The Sr^2+^ enrichment in *P. cathayana* female seedlings was higher than that in male seedlings, while the opposite was true for *S. babylonica*. *P. cathayana* and *S. babylonica* seedlings exhibited heterogeneity in the correlation patterns of Sr^2+^ enrichment (Figure 1C,D). With increasing Sr^2+^ concentration, the enrichment concentration of Sr^2+^ in female seedlings of both plants was positively correlated with the stress concentration of Sr^2+^ (*p* < 0.05), and the enrichment concentration of Sr^2+^ increased significantly in both plants, 3.92 times and 1.96 times, respectively, compared with the low concentration of Sr^2+^ complex stress. The enrichment of Sr^2+^ in male seedlings under combined stress was highly significantly negatively correlated with low concentrations (*p* < 0.01), indicating that diesel had an antagonistic effect on the uptake of low concentrations of Sr^2+^ by male seedlings and was more tolerant to the stress; it was positively correlated with high concentrations of stress (*p* < 0.05), and diesel had a synergistic effect on high concentrations of Sr^2+^, promoting the uptake of Sr^2+^ by male seedlings.

**Figure 2:**
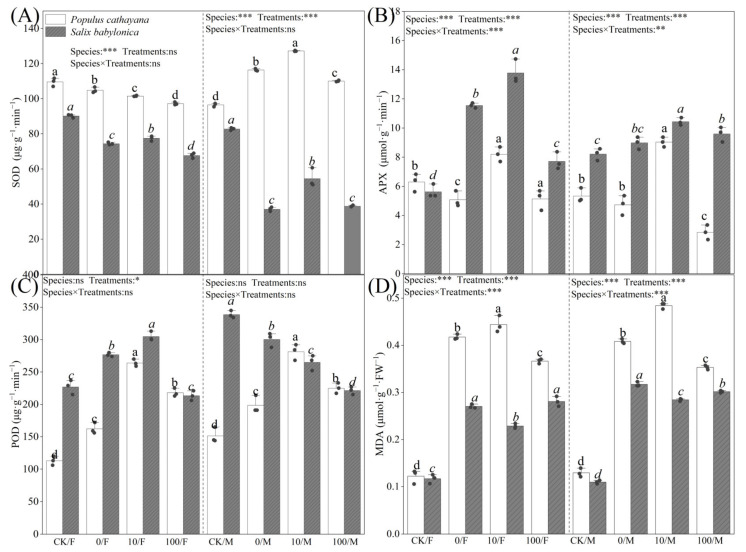
Activities of SOD (**A**), APX (**B**), POD (**C**), and contents of MDA (**D**) of *P. cathayana* and *S. babylonica* under different treatments. Values are expressed as means ± SE (*n* = 5). Values followed by the same letter in the same column are not significantly different (*p* < 0.05). ***: *p* < 0.001; **: *p* < 0.01, *: *p* < 0.05.

The data in Table 1 showed the transfer ability of Sr^2+^ from root to shoot and leaf and the removal efficiency of soil Sr^2+^ by the plant. At low concentrations of Sr^2+^ complex stress, (L/R) and (S/R) transport coefficients were significantly higher in male seedlings of *P. cathayana* than in female seedlings, 1.68 and 15.52 times higher, respectively; female seedlings of *S. babylonica* (L/R) were higher than male seedlings, while (S/R) was the opposite. At high concentrations of Sr^2+^ complex stress, (L/R) and (S/R) transport coefficients were significantly lower in male than in female poplar seedlings, and (L/R) and (S/R) transport coefficients decreased 2.52 times and 20.25 times, respectively, compared with low concentrations; (L/R) and (S/R) transfer coefficients were significantly higher in *S. babylonica* seedlings (L/R: 1.76 times for females and 6.49 times for males; S/R: 5.10 times for females and 2.64-fold for males) (Figure 2A–D). For both species, the females performed higher removal ability of Sr^2+^ from soil to plant than the males at low Sr^2+^ combined stress (*p* < 0.05). Regarding the high Sr^2+^ combined stress, the females of *S. babylonica* exhibited higher removal efficiency than males (*p* < 0.05), contrasting with the *P. cahtayana*. The species *S. babylonica* in general showed better potential removal efficiency than *P. cathayana*.

### 2.2. Gas Exchange Parameters of P. cathayana and S. babylonica

Single diesel stress inhibited transpiration rate (E), stomatal conductance (Gs), and photosynthetic rate (A), and increased intercellular CO_2_ concentration (Ci) in *P. cathayana* and *S. babylonica* seedling leaves of both sexes after 30 d. All gas exchange parameters were reduced in *P. cathayana* male seedlings, indicating that they were mainly influenced by the limitation of stomatal factors; *P. cathayana* seedlings were lower than *S. babylonica* seedlings, except for E, which was higher than *S. babylonica* seedlings (Table 2). Low concentrations of Sr^2+^ complex stress increased Ci and E content, decreased Gs and A content in *P. cathayana* seedlings, and increased Ci, Gs, and A content in *S. babylonica* seedlings. With increasing Sr^2+^ stress concentration, the transpiration rate E of *P. cathayana* seedlings was significantly reduced, and the contents of Gs and A increased. The gas exchange parameters of *S. babylonica* seedlings decreased, except for the increase in Ci content. High Sr^2+^ stress damages the photosynthetic system of *S. babylonica*, which affects the timely conversion of CO_2_ into sugars. *P.* cathayana and *S. babylonica* male seedlings were higher than female seedlings for all gas exchange parameters and were more tolerant than female seedlings. As the treatment time was extended to 60 d, single and combined diesel treatments increased Ci and E and decreased Gs and A in *P. cathayana* and *S. babylonica*, enhancing pollution toxicity to the plants.

### 2.3. Antioxidant Enzymes in P. cathayana and S. babylonica

Figure 2A–D shows the changes in the antioxidant systems of *P. cathayana* and *S. babylonica* after 60 d of stress treatment. Under single diesel stress, POD activity and MDA content of *P. cathayana* seedlings were significantly increased, SOD and APX activities of female seedlings were reduced, and changes in male enzyme activities were opposite to those in females. The SOD activity of *S. babylonica* seedlings decreased, and their APX activity and MDA content increased significantly. The POD and APX activities and MDA contents of *P. cathayana* seedlings were increased considerably under the low concentration of Sr^2+^ complex stress compared with that of diesel alone, while the SOD and APX activities of *S. babylonica* seedlings were significantly increased, while the MDA contents were reversed. The POD activities of *S. babylonica* seedlings showed gender differences, with the POD activities of female seedlings increasing while those of male seedlings decreased. As the Sr^2+^ stress concentration increased, the MDA content of *S. babylonica* seedlings increased significantly, and the antioxidant enzyme activities of *P. cathayana* and *S. babylonica* seedlings decreased significantly but were still higher than those of the control group, indicating that the complex stress caused damage to the plant antioxidant system.

### 2.4. Soluble Matter in P. cathayana and S. babylonica

**Figure 3:**
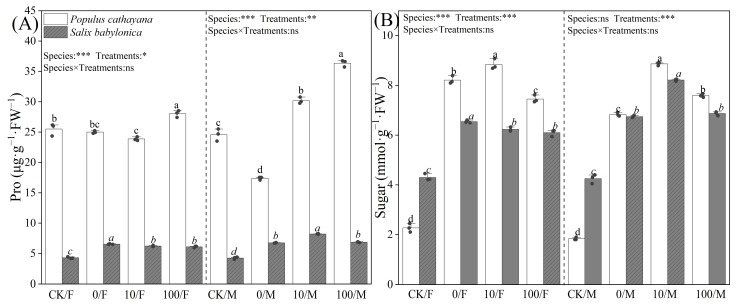
Contents of proline (**A**) and sugar (**B**) of *P. cathayana* and *S. babylonica* under different s treatments. Values are expressed as means ± SE (*n* = 5). Values followed by the same letter in the same column are not significantly different (*p* < 0.05). ***: *p* < 0.001;**: *p* < 0.01, *: *p* < 0.05.

Proline (Pro) and soluble sugar content varied in *P. cathayana* and *S. babylonica* seedlings. Single diesel stress had no significant effect on the Pro of *P. cathayana* females, but the Pro content of male seedlings decreased by 29.28%. The Pro of *S. babylonica* seedlings increased significantly, but the Pro content of *P. cathayana* was much higher than that of *S. babylonica*, and the Pro content of *P. cathayana* female and male seedlings was 3.82 and 2.58 times higher than that of *S. babylonica* females and males, respectively (Figure 3). Soluble sugar content was significantly increased in *P. cathayana* seedlings and *S. babylonica* seedlings (*P. cathayana* females: 2.61 times, *P. cathayana* males: 2.72 times, *S. babylonica* females: 52.20%, *S. babylonica* males: 58.58%) (Figure 3). Under compound stress, with increasing Sr^2+^ concentration, the Pro of *P. cathayana* male seedlings showed a dose-dependent relationship with increasing Sr^2+^ concentration, and the Pro of *P. cathayana* female seedlings increased significantly under high Sr^2+^ stress. However, it had less of an effect on *S. babylonica* seedlings. Compound stress significantly increased the soluble sugar content of the seedlings of both plants compared with that of the control. However, the Pro and soluble sugar content of *P. cathayana* seedlings was higher than those of *S. babylonica* seedlings.

### 2.5. Soil Enzyme Activities of P. cathayana and S. babylonica

**Figure 4:**
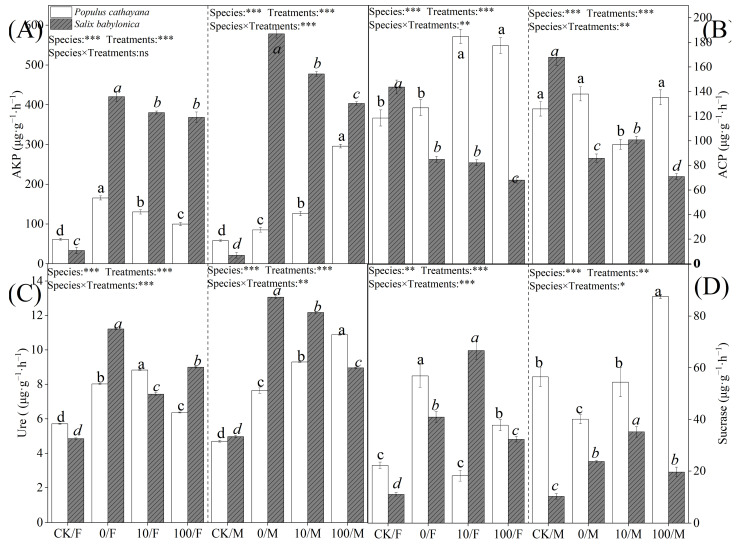
Activity of AKP (**A**), ACP (**B**), Ure (**C**), and sucrase (**D**) in *P. cathayana* and *S. babylonica* with different treatments. Values are expressed as means ± SE (*n* = 5). Values followed by the same letter in the same column are not significantly different (*p* < 0.05). ***: *p* < 0.001;**: *p* < 0.01, *: *p* < 0.05.

Soil enzyme activity also differed in response to stress. Single diesel stress significantly increased alkaline phosphatase (AKP) and Ure activities in soils of *P. cathayana* and *S. babylonica* seedlings. The changes in soil enzyme activities of *S. babylonica* seedlings were more significant than those of *P. cathayana* seedlings, with AKP activity increasing 11.64 times and 26.12 times and Ure activity growing 1.31 times and 1.63 times in soils of *S. babylonica* male and female seedlings, respectively; ACP activity did not change significantly in *P. cathayana* seedlings, while ACP activity decreased significantly in *S. babylonica* seedlings; sucrase (sucrase) activity decreased in the soil of *P. cathayana* male seedlings, and the opposite change was observed in other seedlings (Figure 4). Enzyme activities in *P. cathayana* and *S. babylonica* soils under complex stress conditions were significantly higher than in the control. With the increase of Sr^2+^ complex stress, AKP and Ure activities in the soils of female seedlings of Cyprus and male seedlings of *S. babylonica* decreased significantly, while the changes of enzyme activities in the soils of male seedlings of Cyprus were reversed; ACP activities in the soils of male seedlings of Cyprus increased significantly, while the changes in the soils of female seedlings were not significant; the sucrase activity was significantly increased in *P. cathayana* seedlings; ACP and sucrase activities were significantly decreased in the soil of *S. babylonica* seedlings.

### 2.6. Correlation Analysis

**Figure 5:**
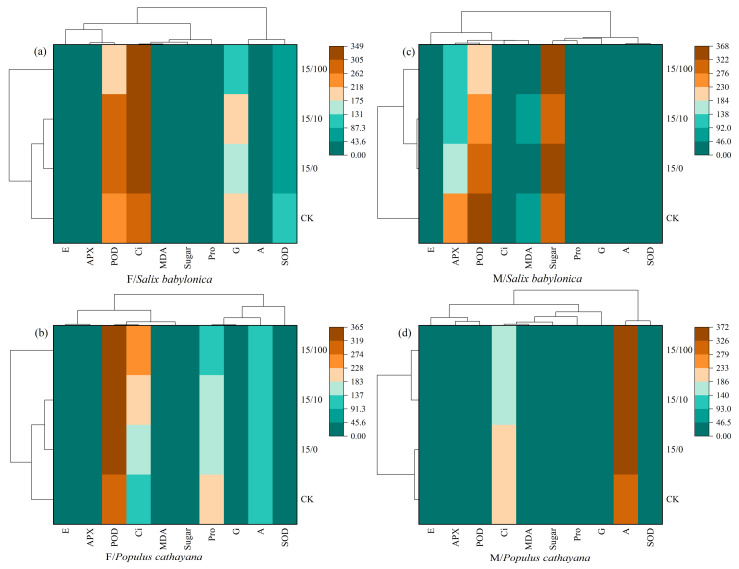
Correlation analysis of each index under different treatments of female (**a**) and male (**c**) for *S. babylonica*, and female (**b**) and male (**d**) for *P.cathayana*.

A heat map was used to further compare the effects of different stress conditions on different plant species and sexes. At single and compound stresses, *S. babylonica* female seedlings and *P. cathayana* female seedlings were strongly correlated with Ci and POD (Figure 5a,b). As Sr^2+^ stress concentration increased, the Ci correlation increased in *S. babylonica* and *P. cathayana* seedlings, the POD correlation weakened in *S. babylonica*, and the POD correlation increased in *P. cathayana*. *S. babylonica* male seedlings were strongly correlated with POD and soluble sugar, and *P. cathayana* male seedlings were most strongly correlated with A (Figure 5c,d). Based on the cluster analysis of different stress conditions, the treatments could be classified into three types. *S. babylonica* and *P. cathayana* female seedlings were classified equally, and different plants of the same sex had similar response mechanisms to the stress conditions. Male seedlings of both plants were divided into two species, indicating that male seedlings were significantly affected by the treatment and could quickly adapt to changes in response to environmental conditions.

## 3. Materials and Methods

### 3.1. Experiment Design

Plant samples from species of *P. cathayana* and *S. babylonica* were collected from the campus experimental garden of Southwest University of Science and Technology, Sichuan Province, China. After growing for about a month, healthy cuttings with approximately uniform size were selected to be planted in 5 L plastic seedling pots filled with homogenized test soil. The properties of the soil used were pH 7.2, soil electrical conductivity (EC) 1.25 ms, 71 mg·kg^−1^ of organic carbon, 86 mg·kg^−1^ of nitrogen, 84 mg·kg^−1^ of potassium, 61 mg·kg^−1^ of phosphorus, 37.2% sand, 21.7% silt, and 3.4% clay. The plants were then grown in a semi-greenhouse on campus with 20.0–34.0 °C and relative humidity of 50–80%.

Eight treatments were established for *P. cathayana* and *S. babylonica* male and female seedlings: control group (CK/F and CK/M: no diesel and no Sr^2+^ applied for females and male trees, respectively), diesel stress group (0/F and 0/M: only 15 mg Kg^−1^ diesel applied for female or male trees, respectively), combined diesel and low Sr^2+^ stress group (10/F and 10/M: 15 mg Kg^−1^ diesel + 10 mg Kg^−1^ Sr^2+^ applied for female or male trees, respectively), and combined diesel and high Sr^2+^ stress group (100/F and 100/M: 15 mg Kg^−1^ diesel + 100 mg Kg^−1^ Sr^2+^ applied for female or male trees, respectively), with five replicate pots for each treatment. Forty cuttings of each sex for every species were randomly arranged to stress for two months. The diesel fuel applied was No. 0# diesel from PetroChina Co., Ltd., and the Sr^2+^ applied was from strontium chloride hexahydrate (SrCl_2_.6H_2_O, Sigma-Aldrich). The pollutants were mixed and pre-equilibrated with treated soil for at least two weeks to ensure a uniform distribution.

### 3.2. Measurement Methods

#### 3.2.1. Determination of Sr Content in Plant Tissues

Samples of each tissue were collected, killed at 105 °C for 30 min, and then dried at 70 °C to a constant weight. The dried roots, stems, and leaves were collected and ground into powder. Then, 0.2 g of dried sample were separated, adopting a graphite furnace wet nitrification, adding 15 mL of mixed acid (volume ratio of nitric acid: perchloric acid is 3:1), put into a graphite furnace, and heated at 200 °C until the digestion solution was clear and bright. Then, 10 mL of deionized water was added, and heating was applied until no smoke appeared. The remaining digestion solution was fixed at 50 mL, shaken well, and left to stand. The supernatant was collected for determination. Three replicates of each treatment were measured, and the content of Sr in each tissue was determined by atomic absorption spectrometry (PE 900T, Perkin Elmer Company, USA). The analysis conditions included a wavelength of 460.7 nm, a lamp current of 20 mA, a spectral passband of 0.2 nm, combustion gas as air at a rate of 16 L min^−1^, and acetylene as gas at a 7.8 L min^−1^.

#### 3.2.2. Gas Exchange Measurements

The gas exchange parameters of leaves, including transpiration rate (E), stomatal conductance (Gs), net photosynthetic rate (A), and intercellular CO_2_ concentration (Ci), were measured from 9:00 to 11:00 a.m. using a GFS-3000 (Walz Co., Ltd., Effeltrich, Germany) with the light intensity at 800 μmol m^−2^ s^−1^ and the leaf chamber temperature at 25 °C by thermoelectric cooler. Five cuttings from each treatment were selected for measuring.

#### 3.2.3. Determination of Antioxidant Enzyme Activity and Malondialdehyde

The superoxide dismutase (SOD) activity was according to the nitrogen blue tetrazolium method, and the peroxidase (POD) activity was determined by the guaiacol method described by Guo et al. [19], and the result was expressed by μg·g^−1^·min^−1^. The ascorbate peroxidase (APX) activity was determined as described by Mishra et al. [20], and the result was expressed by μmol·g^−1^·min^−1^. The malondialdehyde (MDA) content was determined using the thiobarbiturate method described by Yang and Gao [21], and the result was expressed by μmol·g^−1^·FW.

#### 3.2.4. Determination of Free Proline and Soluble Sugar Contents

Proline content was determined using the acidic ninhydrin method. The plant tissues were homogenized in 5 mL of 3% for 10 min at 100 °C. After centrifuging at 3000× *g* for 10 min, glacial acetic acid and ninhydrin were added to the supernatant. Then, free proline was extracted with toluene and measured by spectrophotometer (UV, Unico Instrument, Shanghai, China). The soluble sugar content was determined using the phenol–sulfuric acid method with soluble sugar detection kit (G0501F, Suzhou Grace Biotechnology Co., Ltd., Suzhou, China).

#### 3.2.5. Measurement of Soil Enzyme Activity

Referring to Lei et al. [22], determination of the soil sucrase activity was determined by the 3,5-dinitrosalicylic acid method. Soil urease (Ure) activity was determined using the sodium phenol–sodium hypochlorite colorimetric method. Soil acid phosphatase (ACP) and alkaline phosphatase (AKP) activities were determined using a colorimetric method with sodium benzodiphosphate.

### 3.3. Data Analysis

Data are expressed as the average standard error (SE) of independent measurements. Differences between groups were analyzed using one-way analysis of variance (ANOVA). The Origin 2022 software was used for basic mapping and information processing.

## 4. Discussion

Soil pollution caused by the combination of Sr and diesel fuel presents a considerable challenge for plants. The uptake capacity of pollutants by plant roots and the enrichment capacity of plants are the main phytoremediation capabilities. The uptake capacity of Sr^2+^ varies among tissues, and the root system is vital for plant uptake and metabolism [23]. Our results, in accordance with previous works that plants accumulate pollutants in the root system, which reduces the migration of metals to the stem and leaves and helps reduce the toxic effects on the aboveground parts [24]. In addition, for *P. cahtayana* in the combined stress, the male seedlings preferred to transfer Sr^2+^ to aboveground parts to mitigate the toxic effects at low Sr^2+^ concentration but mainly accumulated Sr^2+^ in the root to alleviate the stress at high Sr^2+^ concentration. For *S. babylonica,* the male performed higher enrichment than the female, indicating that *S. babylonica* males have a stronger enrichment capacity and are potential plants for remediation of nuclide metal and PAHs pollution. The results also showed that at high concentrations of Sr^2+^ complex stress, diesel had an antagonistic effect on Sr^2+^ enrichment in male seedlings, whereas it had a synergistic effect on Sr^2+^ enrichment in female seedlings. Synergistic effects of complex stress on plant growth have been reported previously [25].

In plants, photosynthesis is limited by stomatal and non-stomatal factors [26]. According to Faquhar, the intercellular CO_2_ concentration Ci is an important parameter for determining the decrease in the photosynthetic rate [27]. In this study, the photosynthetic rates of both plants were mainly limited by non-stomatal factors under a single diesel stress, and the diesel-induced damage to the photosynthetic machinery of the leaf flesh prevented the timely conversion of CO_2_ to organic matter in the leaf flesh cells, leading to an increase in intercellular CO_2_ concentration. The reduction in the photosynthetic rate of *P. cathayana* male seedlings was mainly caused by the decrease in stomatal factors, indicating that the stress treatment caused less damage to the photosynthetic system of *P. cathayana* male seedlings, which were poisoned and involved in regulating the metabolism of physiological functions in the form of transpiration and were more tolerant to diesel. The combination of Sr^2+^ stress at low concentrations showed an antagonistic effect on *S. babylonica* seedlings, reducing the toxicity of a single stress, similar to the results of Li et al. [28]. In contrast, it showed a synergistic effect on *P. cathayana* seedlings. Compared to the CK group, the photosynthetic rate of *P. cathayana* and *S. babylonica* seedlings was significantly reduced due to non-stomatal factors, and the physiological function of chloroplasts was disrupted, which caused significant inhibition and damage to photosynthesis and the physiological activities of the plants. The results showed that high concentrations of Sr^2+^ complex stress caused strong toxic effects in both plants; however, male seedlings were more tolerant of the toxic effects than female seedlings in both species.

Plants respond to abiotic stress by producing antioxidant enzymes, considered important defense mechanisms against oxidative stress caused by PAHs. MDA content is often used to evaluate the level of damage caused by lipid peroxidation under heavy metal stress [29]. Under a single diesel stress, the plant resistance mechanism was stimulated, thus reducing the damage caused by ROS in seedling cells. *P. cathayana* seedlings were more damaged than *S. babylonica* seedlings, probably because MDA binds to proteins and enzymes on the cellular biofilm of *P. cathayana* seedlings, causing cross-linking within and between protein molecules and resulting in the inability of the cellular membrane system to perform normal physiological functions, similar to the results of Cheng et al., who studied oxidative stress in ryegrass under combined PAH and Cd soil conditions [30]. The increased antioxidant capacity and decreased MDA content of *P. cathayana* and *S. babylonica* seedlings under low concentrations of Sr^2+^ complex stress compared to single stress indicated that low concentrations of Sr^2+^ had a mitigating effect on diesel-induced pollution toxicity. Based on the results of Feria’s study, it was hypothesized that the presence of diesel alters the uptake process of Sr^2+^ in plants and affects the tricarboxylic acid cycle and oxidative stress [31]. Prolonged exposure to PAH stress induces the activity of antioxidant enzymes, leading to a decrease in MDA content produced by lipid peroxidation [32]. In response to pollution stress, the antioxidant enzyme system of *P. cathayana* seedlings showed the most significant change in POD activity, indicating that *P. cathayana* mainly responded to oxidative stress by increasing POD activity, whereas *S. babylonica* mitigated oxidative damage by increasing APX. The antioxidant systems of male seedlings of *P. cathayana* and *S. babylonica* were more stable than those of female seedlings, and male seedlings were more resistant to oxidative stress.

Soil enzymes are a class of biologically active substances that participate in biochemical processes, such as nutrient cycling, organic matter mineralization, and pollutant degradation in the soil [33]. Under stress conditions, soil enzyme activities were significantly higher in both plants than in the control group, showing an activation effect in which microorganisms were able to use diesel as a source of carbon, nitrogen, and energy to stimulate their growth, thus activating soil enzyme activities [34]. The effects of Sr^2+^ and diesel pollution on soil enzyme activity were antagonistic. Shen et al. demonstrated that the combined contamination of heavy metals and PAHs showed an antagonistic effect on the Ure activity of Zn and benzo(a)pyrene [35]. The coexistence of organic contaminants and heavy metals in the soil environment may affect the transformation and metabolic pathways of contaminants in the environment by competing for sorption sites, forming heavy metal–organic contaminant complexes, or altering soil properties. Soil enzyme activity is influenced by a variety of environmental factors, and different plant species and sexes differ in their growth processes, with different active substances, such as organic acids and growth hormones secreted by the root system, producing differences in their impact on microbial growth.

The positive correlation between Ci and Sr^2+^ stress concentration in female *S. babylonica* and *P. cathayana* seedlings indicated that increasing Sr^2+^ concentration had significant effects on CO_2_ fixation and sugar synthesis in female seedlings. Combined cluster analysis showed that female *P. cathayana* and *S. babylonica* seedlings had similar defense mechanisms in response to different stresses, whereas male seedlings of different plants differed more in their defense. With limited resources, female trees tended to conserve energy for reproduction, whereas male trees devoted more resources to nutritional growth and stress resistance [36]. Male trees of different plants experience abiotic stresses, such as UV radiation, organic matter, and heavy metal pollution, and are more resistant to these stressors than females [37]. Consequently, different defense mechanisms have evolved to cope with various stresses, and plants of different sexes exhibit different physiological and biochemical responses to environmental stresses.

## 5. Conclusions

Our results show that *P. cathayana* and *S. babylonica* have some tolerance and Sr^2+^ enrichment ability to soil Sr^2+^ and diesel pollution stress. Male seedlings have higher enrichment ability and tolerance than females, and *S. babylonica* has more tolerance to diesel and Sr^2+^ stress and the ability to remediate polluted soil. This is attributed to Sr^2+^ accumulation, tolerance, and detoxification strategies of different plant species and sexes. Diesel pollution alone inhibited the growth of both species, reduced the photosynthetic rate, activated antioxidant enzyme activity to mitigate oxidative damage to the cell membrane system from elevated MDA content, and increased soluble matter content and soil enzyme activity in response to stress damage. There were differences in the stress mechanisms of low concentration of Sr^2+^ complex stress on *P. cathayana* and *S. babylonica* seedlings compared to single diesel stress. *S. babylonica* seedlings were mainly enriched in the roots with higher enrichment capacity than *P. cathayana* seedlings, but the transport coefficient was lower than that of *P. cathayana* seedlings, and the detoxification of Sr^2+^ was mainly due to the exocytosis mechanism. *P. cathayana* males were less enriched than female seedlings, but the transport coefficient was larger, the detoxification mechanism was based on accumulation and compartmentalization, and their tolerance was stronger than that of female seedlings. Low concentrations of Sr^2+^ alleviated the toxicity of diesel fuel in plants, and the antioxidant system of male seedlings was more stable than that of female seedlings. ACP enzyme activity was higher in the soil of *P. cathayana* seedlings, whereas it was dominated by AKP and urea in the soil of *S. babylonica* seedlings. The combined stress of high Sr^2+^ concentration and diesel had a synergistic effect, enhancing the toxic effects on *P. cathayana* and *S. babylonica* seedlings, whereas the toxicity to the plants became stronger as the stress time increased. The results of this study provide a reference for the phytoremediation and treatment of nucleophilic Sr^2+^ and diesel fuel complex-contaminated soil, and woody phytoremediation has broad application prospects.

## Figures and Tables

**Table 1:** Transport coefficient and removal efficiency of *P. cathayan* and *S. babylonica*.

	*Populus Cathayana*				*Salix Babylonica*			
	10/F	100/F	10/M	100/M	10/F	100/F	10/M	100/M
S/R	0.298 ± 0.015 ^e^	0.4420.002 ^d^	4.930 ± 0.019 ^a^	0.232 ± 0.0107 ^f^	0.134 ± 0.006 ^g^	0.818 ± 0.014 ^b^	0.150 ± 0.005 ^g^	0.546 ± 0.001 ^c^
L/R	0.740 ± 0.033 ^d^	0.662 ± 0.008 ^d^	1.981 ± 0.065 ^a^	0.562 ± 0.019 ^e^	0.382 ± 0.009 ^f^	1.061 ± 0.017 ^c^	0.213 ± 0.007 ^g^	1.595 ± 0.005 ^b^
RE (%)	3.602 ± 0.027 ^b^	1.427 ± 0.056 ^d^	1.167 ± 0.072 ^e^	0.792 ± 0.031 ^f^	6.032 ± 0.061 ^a^	1.389 ± 0.013 ^d^	1.091 ± 0.008 ^e^	2.341 ± 0.011 ^c^

Note: For *P. cathayana* or *S. babylonica*, S/R represents Sr^2+^ content in shoot/Sr^2+^ content in root transfer coefficient. L/R represents Sr^2+^ content in leaf/Sr^2+^ content in root transfer coefficient. RE expressed as Sr^2+^ contents in plant/Sr^2+^ content in soil for each pot to represent Sr^2+^ removal efficiency from soil. Different lowercase letters indicate the different treatments within the same sample site (*p* < 0.05) (mean ± SD, *n* = 5).

**Table 2:** Photosynthetic parameters of *P. cathayan* and *S. babylonica*.

Sexuality	Treatment Group	*Populus Cathayana*				*Salix Babylonica*			
		Ci	Gs	E	A	Ci	Gs	E	A
Female	CK	296.510 ± 5.094 ^e^	184.134 ± 6.933 ^c^	2.326 ± 0.111 ^c^	8.127 ± 0.413 ^c^	292.801 ± 3.892 ^e^	194.021 ± 8.727 ^b^	2.085 ± 0.098 ^c^	8.943 ± 0.249 ^c^
	15/0	328.102 ± 5.215 ^d^	175.936 ± 9.710 ^d^	2.262 ± 0.039 ^d^	7.751 ± 0.200 ^d^	333.680 ± 3.965 ^c^	157.240 ± 12.171 ^c^	2.584 ± 0.098 ^a^	7.472 ± 0.330 ^d^
	15/10	364.890 ± 4.836 ^b^	117.959 ± 9.149 ^g^	2.614 ± 0.095 ^a^	6.383 ± 0.370 ^f^	327.990 ± 2.895 ^c^	199.630 ± 5.311 ^b^	2.044 ± 0.122 ^c^	8.915 ± 0.270 ^c^
	15/100	360.880 ± 3.839 ^b^	161.945 ± 9.938 ^e^	2.094 ± 0.090 ^e^	7.131 ± 0.281 ^e^	348.520 ± 2.895 ^b^	129.050 ± 7.368 ^d^	1.758 ± 0.093 ^d^	6.389 ± 0.326 ^f^
Male	CK	300.920 ± 4.263 ^e^	200.170 ± 9.850 ^a^	2.591 ± 0.091 ^b^	9.316 ± 0.274 ^b^	280.400 ± 5.740 ^e^	233.480 ± 6.466 ^a^	1.904 ± 0.069 ^c^	10.588 ± 0.287 ^a^
	15/0	333.370 ± 6.152 ^d^	196.741 ± 7.408 ^a^	2.111 ± 0.083 ^e^	9.998 ± 0.334 ^a^	330.00 ± 5.990 ^c^	150.630 ± 8.116 ^c^	2.290 ± 0.098 ^b^	8.871 ± 0.323 ^c^
	15/10	346.410 ± 3.983 ^c^	160.459 ± 9.050 ^e^	2.688 ± 0.049 ^a^	8.478 ± 0.381 ^c^	314.25 ± 2.022 ^d^	127.440 ± 8.291 ^d^	1.981 ± 0.094 ^c^	9.923 ± 0.382 ^b^
	15/100	371.970 ± 4.698 ^a^	140.609 ± 6.994 ^f^	2.254 ± 0.083 ^d^	7.147 ± 0.292 ^e^	367.110 ± 3.688 ^a^	104.860 ± 6.895 ^e^	1.416 ± 0.111 ^e^	7.014 ± 0.411 ^e^

Note: CK: no diesel or Sr^2+^ applied; 15/0: 15 mg Kg^−1^ diesel + 0 mg Kg^−1^ Sr^2+^; 15/10: 15 mg Kg^−1^ diesel + 10 mg Kg^−1^ Sr^2+^; 15/100: 15 mg Kg^−1^ diesel + 100 mg Kg^−1^ Sr^2+^. Different lowercase letters indicate the different treatments within the same sample site (*p* < 0.05) (mean ± SD, *n* = 5).

## Data Availability

The data presented in this study are available upon reasonable request from the corresponding authors.
